# Editorial: Cardiac rhythmology case reports: Abnormal ECG and beyond

**DOI:** 10.3389/fcvm.2022.919117

**Published:** 2022-08-12

**Authors:** Chun-Ka Wong, Hung-Fat Tse

**Affiliations:** ^1^Cardiology Division, Department of Medicine, Li Ka Shing Faculty of Medicine, The University of Hong Kong, Hong Kong, Hong Kong SAR, China; ^2^Hong Kong-Guangdong Stem Cell and Regenerative Medicine Research Centre, The University of Hong Kong and Guangzhou Institutes of Biomedicine and Health, Guangzhou, China; ^3^Center for Translational Stem Cell Biology, Hong Kong, Hong Kong SAR, China; ^4^Heart and Vascular Center, The University of Hong Kong-Shenzhen Hospital, Shenzhen, China

**Keywords:** electrocardiogram, left bundle branch pacing, atrial tachycardia, Guillain Barre Syndrome, pancreatitis

A 12-lead surface electrocardiogram (ECG) is an essential initial investigation for most cardiac conditions, both for establishing the diagnosis and facilitating management. Moreover, ECG also provides a wide array of clinical information, from physiological condition and homeostasis to active and past cardio-pulmonary pathologies. Clinicians might occasionally be tempted to solely rely on ECG findings for making diagnoses. Nevertheless, it is essential to go beyond ECG findings and perform a holistic clinical assessment, including detailed history taking, meticulous physical examination, and adjunct investigation, including blood tests, echocardiography, and other imaging modalities, as well as invasive investigations, to guide proper clinical management. The approach of going beyond ECG abnormality identification to whole-patient care was well-exemplified by the authors who contributed to this case report Research Topic on interesting ECG findings in *Frontier in Cardiovascular Medicine*.

We curated two illustrative case reports related to pre-procedural planning and post-procedural complications with intriguing ECG findings. Luo et al. showcased the management of a pediatric patient with refractory atrial tachycardia despite anti-arrhythmic medications, and complicated with tachycardia-mediated cardiomyopathy. Pre-procedural ECG suggested that the atrial tachycardia originated from the right atrial appendage with a negative P wave in lead V1 and a positive towering P wave in the inferior leads. Using a three-dimensional electroanatomical mapping system, the atrial tachycardia foci was indeed localized at the apex of the right atrial appendage. As the atrial tachycardia could not be terminated after extensive catheter-based endocardial radiofrequency ablation, the authors switched to a thoracoscopic approach and performed clamp radiofrequency ablation using bipolar radiofrequency ablation forceps to the base of right atrial appendage. Atrial tachycardia was successfully terminated with no recurrence during the next 3 months of follow up. With resolution of atrial tachycardia accompanied by guideline-directed medical therapy, the left ventricular systolic function of this patient improved from 31 to 60%. In another interesting case report, Zheng et al. reported a rare post-procedural complication after left bundle branch area pacing (LBBP). Since the introduction of LBBP in 2017 (1), this novel pacing technique has been increasingly utilized as an alternative approach to his bundle pacing for physiologic conductive system pacing, as LBBP has a high implant success rate and preserves left ventricular function compared to conventional right ventricular sites pacing in recent observational studies (2, 3). Nonetheless, there are limited data on the short- and long-term safety of this novel LBBP approach. Zheng et al. reported a patient with chest pain, non-specific ST-segment depression on ECG, and markedly elevated troponin level 1 hour after LBBP. Careful examination with transthoracic echocardiography by the authors revealed swollen interventricular septum and the computer tomography angiography (CTA) confirmed the presence of a septal hematoma. It was postulated that the hematoma resulted from inadvertent injury to one of the septal perforating arterial branches. The patient was conservatively treated, and a serial imaging 1 month later showed resolution of the hematoma.

It is well-known that patients with acute medical or surgical illnesses may also manifest abnormal ECG findings despite having no definite cardiac pathology. Some of these ECG changes may mimic acute coronary syndrome; for instance, ST segment changes were not uncommon among patients with ischemic or hemorrhagic stroke (4). It has been proposed that those ECG abnormalities can be mediated by changes in autonomic nervous system activity, secondary to underlying systemic or intracranial pathologies, without necessarily implicating underlying cardiac disease. It may sometimes be challenging for clinicians to accurately determine whether acute cardiac complications are present that warrant urgent investigation and intervention. Cao et al. reported a middle-aged woman with Guillain Barre Syndrome, who complained of multiple pain over chest, back, and limbs. ECG showed transient T-wave inversion over lead I, aVL, and from V2 to V4. Cardiac injury biomarkers and echocardiography were both normal. Underlying coronary artery disease was initially considered less likely as the patient had no known cardiovascular risk factors, and Guillain Barre Syndrome was known to be associated with ECG changes. In a retrospective study involving 96 patients with Guillain Barre Syndrome, ECG abnormalities were present in 50% of the patients, with 12.5% having ST-T changes (5). Despite the patient's relatively low probability of having coronary artery disease, the authors proceeded to arrange a CT coronary angiogram based on clinical suspicions, as the patient had recurrent chest pain. A critical stenosis was found to be present in the left anterior descending artery, and the patient subsequently underwent coronary revascularization procedure. In another case report, Long et al. described an elderly woman presented with acute pancreatitis with diffuse abdominal tenderness. She had no chest pain, but ECG showed ST-segment elevation over inferior leads. It was uncertain whether the ECG changes reflected true coronary pathology or were merely the ECG changes associated with acute pancreatitis. In a small case series, it was reported that up to 55% of patients with pancreatitis had abnormal ECG findings (6), and ECG findings mimicking ST-elevated myocardial infarction have also been reported in case reports as well (7). It was critical to distinguish between true acute coronary syndrome and non-specific ECG changes secondary to pancreatitis because it had significant implications in downstream management strategies. Acute coronary syndrome would require prescription of anti-thrombotic mediations, which confers additional bleeding risk to the patient with pancreatitis. On the other hand, missing the opportunity to arrange timely interventional or pharmacological therapy for acute coronary syndrome would compromise cardiac outcome. The authors eventually arranged echocardiography and CTA, which confirmed that the patient had critical stenosis over the left anterior descending artery and right coronary artery.

We congratulate the above authors for publication of those enlightening case reports, which demonstrated the importance of careful analysis and interpretation of ECG, which subsequently resulted in appropriate further investigation and treatment of underlying conditions.

## Author contributions

C-KW and H-FT wrote manuscript and revised the manuscript critically for important intellectual content. Both authors have read and approved the final version of the manuscript to be published.

## Conflict of interest

The authors declare that the research was conducted in the absence of any commercial or financial relationships that could be construed as a potential conflict of interest.

## Publisher's note

All claims expressed in this article are solely those of the authors and do not necessarily represent those of their affiliated organizations, or those of the publisher, the editors and the reviewers. Any product that may be evaluated in this article, or claim that may be made by its manufacturer, is not guaranteed or endorsed by the publisher.
